# Disparities in human papillomavirus vaccination uptake across the intersection of disability and sexual orientation

**DOI:** 10.1007/s10552-025-02025-z

**Published:** 2025-06-25

**Authors:** Amarachukwu F. Orji, Gilbert Gimm, Tarang Parekh, Rodman Turpin, Carolyn Drews-Botsch

**Affiliations:** 1 Department of Global and Community Health, College of Public Health, George Mason University, Fairfax, VA USA; 2 Department of Health Administration and Policy, George Mason University, Fairfax, VA USA; 3 Department of Epidemiology, College of Health Sciences, University of Delaware, Newark, DE 19713 USA

**Keywords:** Human papillomavirus vaccination, HPV, Intersectionality, LGB, Disability

## Abstract

**Purpose:**

Although human papillomavirus (HPV) vaccination provides effective primary prevention against cervical cancer, HPV vaccination rates remain low in the U.S. It is unknown whether women with disabilities and/or LGB + women are likely to experience disparities in HPV vaccination uptake.

**Methods:**

We used data from the 2014–2022 Behavioral Risk Factor Surveillance System (*N* = 40,401) to estimate HPV vaccination rates among women aged 18–44 years. Adjusted prevalence ratios (aPRs) were estimated using modified Poisson regression models for four subgroups defined by disability status and LGB + status. Analyses were further stratified by age (18–26 years; 27–44 years).

**Results:**

Overall, only 19% of eligible women had received at least one dose of the HPV vaccine, and 12% had completed the full series. Younger women were more likely than older women to have been vaccinated. Compared with heterosexual women without disabilities, heterosexual women with disabilities, had lower vaccination uptake (heterosexual with disabilities: aPR = 0.95; 95% CI 0.93–0.97; LGB + with disabilities: aPR = 0.97; 95% CI 0.96–0.98), and were 12% less likely to complete the series (heterosexual with disabilities: aPR = 0.88; 95% CI 0.83–0.93).

**Conclusion:**

HPV vaccination rates remain low, representing missed opportunities for primary prevention. Women with disabilities had lower vaccination rates than women without disabilities and were less likely to complete the vaccine series. These findings, combined with age-stratified results, highlight the need for stronger catch-up campaigns and targeted efforts to address structural barriers related to disability, ultimately improving HPV vaccination coverage and reducing the burden of cervical cancer.

## Introduction

Human papillomavirus (HPV) is the most prevalent sexually transmitted infection in the United States [1, 2] affecting approximately one in four sexually active individuals [3]. The lifetime risk of HPV infection is estimated to exceed 50% [4, 5]. While many infections are asymptomatic and resolve naturally within two years, persistent HPV infections can lead to cervical intraepithelial neoplasia and, ultimately, cervical cancer [6, 7]. Each year, nearly 50,000 new cases of HPV-associated cancers are diagnosed in the U.S., including an estimated 13,360 new cases of cervical cancer and 4,320 related deaths projected for 2025 [8]. Over 95% of cervical cancers are attributable to HPV, with 70% caused by HPV types 16 and 18 [9].

The introduction of highly effective HPV vaccines, alongside routine cervical cancer screening, has significantly reduced the burden of HPV-related disease and mortality [10]. The most recent HPV vaccine provides protection against nine HPV types 6, 11, 16, 18, 31, 33, 45, 52, and 58, which are associated with the majority of HPV-related cancers and genital warts [11]. It also has the potential to provide primary prevention for up to 90% of cervical cancers [11]. Initially licensed for individuals aged 9–26, HPV vaccine eligibility has since expanded to include those up to age 45 [9, 10, 12, 13]. In 2019, the Advisory Committee on Immunization Practices (ACIP) recommended routine vaccination at age 9, with a two-dose regimen for those vaccinated before age 15 and a three-dose regimen for those initiating vaccination at age 15 or older [14, 15]. ACIP also recommends catch-up vaccination through age 26, with shared decision-making for those aged 27–45 [12, 14, 15].

Despite the vaccine’s proven efficacy and expanded eligibility, HPV vaccination rates for the general U.S. population remain far below the 80% coverage goal set by Healthy People 2030 [1, 16–18]. Uptake is particularly low among marginalized populations, including women with disabilities and LGB + women [1] both of whom may be at increased risk for HPV infection and cervical cancer [19–22]. Understanding these HPV vaccination disparities is essential to advancing equitable cancer prevention efforts.

Research on HPV vaccine uptake among LGB + women has yielded mixed findings, with some studies indicating lower vaccination rates and others reporting comparable or higher rates among specific subgroups [23]. Similarly, studies on HPV vaccination among individuals with disabilities have been limited by small sample sizes and have primarily focused on adolescents and young adults under age 26 [24]. Both groups encounter distinct yet overlapping barriers to vaccination, including limited healthcare access, provider bias, medical mistrust, limited provider recommendations, transportation difficulties, and accessibility issues within healthcare settings [20, 21, 25–27].

Despite evidence of disparities in HPV vaccination by disability and sexual orientation, few studies have examined how these factors intersect. This gap in knowledge is particularly relevant since disability is more prevalent among LGB + individuals [28–30]. Given that both LGB + individuals and people with disabilities face reduced access to reproductive and preventive healthcare services, and routine gynecologic care [19–25], it is important to examine these groups together. Moreover, because the risk of HPV among sexually active adults is nearly universal, ensuring equitable access to primary prevention for women with disabilities and LGB + women is just as imperative as it is for heterosexual women and those without disabilities [4, 5]. Therefore, understanding how HPV vaccination uptake is affected at the intersection of disability and sexual orientation can help identify targeted interventions to improve access and reduce disparities.

In line with the intersectionality framework, this study examines how disability and sexual orientation, both markers of structural inequities, are associated with HPV vaccination uptake and series completion. Given the differences in HPV vaccination recommendations by age, we analyzed two separate age groups (18–26 vs. 27–44 years). Specifically, this study explores how the combination of these two factors (disability and sexual orientation) contributes to disparities, providing quantitative insight to inform HPV infection and cervical cancer prevention efforts and advance health equity. We hypothesize that LGB + women with disabilities will have lower HPV vaccine initiation and series completion rates than heterosexual women without disabilities, due to compounded structural barriers associated with ableism and heterosexism.

## Methods

### Data and study population

This study used individual-level data from the Behavioral Risk Factor Surveillance System (BRFSS), an annual telephone survey conducted by the Centers for Disease Control and Prevention (CDC) that includes more than 400,000 U.S. residents from all 50 states and all U.S. territories [31]. BRFSS collects state-based data on health behaviors, conditions, and preventive practices among non-institutionalized adults aged ≥ 18 years [32]. This analysis includes data from 2014 to 2022 for the 20 states that administered both the optional Adult HPV Vaccination and Sexual Orientation and Gender Identity (SOGI) modules. The final pooled dataset, which had an average response rate of 47.4%31, allowed for the analysis of four subgroups defined by disability and sexual orientation: (1) heterosexual women without disabilities (reference group), (2) heterosexual women with disabilities, (3) LGB + women without disabilities, and (4) LGB + women with disabilities. Because SOGI module participation varied by state and year, we followed BRFSS guidance for pooled analyses and accounted for this variation by including survey year as a covariate in all models [31]. A complete list of the included states and the corresponding years they participated is provided in Supplementary Table 1.

This analysis was restricted to women eligible for routine and catch-up HPV vaccination, as well as cervical cancer screening. Therefore, we included women aged 18–44 years. The upper age limit accommodates both the BRFSS age categories and the goal of capturing women who would have been up to age 26 in 2006, when the HPV vaccine was first licensed. A total of 77,325 respondents completed both modules; we excluded those outsides the study’s age group (*n* = 20,737), as well as those missing information on HPV vaccination status (*n* = 5,353), disability status (*n* = 1,950), or sexual orientation (*n* = 8,884). The final analytic sample included 40,401 women aged 18–44 years. Throughout this paper, the term “women” refers to participants who self-reported “female” when asked about their sex in BRFSS. The study was deemed exempt by an Institutional Review Board because it used de-identified, secondary data.

## Dependent variable

### HPV vaccination status

The primary outcome of the study was HPV vaccination receipt, which was based on a binary (yes/no) self-reported answer to the following question: “A vaccine to prevent the human papillomavirus or HPV infection is available and is called the cervical cancer or genital warts vaccine, HPV shot [if female ‘GARDASIL or CERVARIX,’ if male ‘GARDASIL’]; Have you ever had the HPV vaccination?” A follow-up question asked, “How many HPV shots did you receive?” (none, partially vaccinated [received greater than or equal to one dose of the series], fully vaccinated [received all three doses]). In this study, we use ‘HPV vaccination receipt’ and ‘HPV vaccination uptake’ interchangeably.

## Primary independent variables

### Dimensions of social position

#### Sexual orientation

Sexual orientation was assessed using responses to: “Which of the following best represents how you think of yourself?” The response options were lesbian or gay; straight, that is, not gay; bisexual; something else; and “I don’t know the answer.” Due to sample-size constraints, we combined lesbian or gay, bisexual, and “something else” into a single category labeled “LGB +.”

#### Disability

Disability was assessed using six standard disability-related questions established by the U.S. Department of Health and Human Services. These questions covered difficulty in hearing, vision, cognition, communication, self-care, and walking or using stairs. Based on these six key questions, a five-category measure of disability was created, grouping respondents into mutually exclusive categories: sensory disability, cognitive disability, physical disability, disabilities in multiple functional areas, and no disability (reference) [19, 33, 34]. Sensory disability was defined as difficulty hearing or seeing; physical disability as difficulty with self-care, walking, or using stairs; and cognitive disability as difficulty with cognition or communication. Respondents reporting challenges in more than one domain were classified as having disabilities in multiple functional areas (multiple disabilities). Those who answered ‘no’ to all six questions were classified as having no disability, while those who answered ‘yes’ to any of the questions were classified as having any disability [19, 33, 34].

## Covariates

Informed by Ronald Andersen’s behavioral model of health service utilization, [35–37] which conceptualizes predisposing, enabling, and need-based factors influencing healthcare behaviors, we considered several demographic, socioeconomic, and healthcare variables as potential confounding variables: age (18–26 years [ref], 27–44 years), race/ethnicity (non-Hispanic White [ref], non-Hispanic Black, Hispanic of any race, and non-Hispanic Other race), marital status (never married [ref], married, divorced/separated/widowed), education (less than high school [ref], high school graduate, some college, college graduate), household income category (< $50,000 [ref], $50,000 or more), geographic region (Northeast [ref], Midwest, South, West), BMI (normal [ref], underweight, obese), health insurance (no [ref], yes), having a regular primary care provider (no [ref], yes), routine health checkup in the past year (no [ref], yes), and current smoking behavior (no [ref], yes).

We used a directed acyclic graph (DAG) to identify a minimal sufficient adjustment set of confounders for estimating the relationship between sexual orientation, disability status, and HPV vaccination. The DAG approach helped determine which variables were necessary to address confounding while avoiding inclusion of variables that might be in the causal pathway. Based on the DAG, we identified age (18–26 years [ref], 27–44 years), race/ethnicity (non-Hispanic White [ref], non-Hispanic Black, Hispanic of any race, and non-Hispanic Other race), and census region as common causes of both sexual orientation and disability status, and therefore necessary confounders [35, 36, 38]. Additionally, we included survey year to account for potential changes in sampling methodology or weighting procedures over time. We excluded the remaining variables because, although they were associated with both the exposures and the outcome, their association was more likely attributable to the impact of a woman’s disability/LGB + status on these characteristics rather than the reverse. Consequently, these variables were deemed to be in the causal pathway rather than true confounders. The DAG was plotted using the DAGitty website, version 3.1 (http://www.dagitty.net/).

### Statistical analysis

We used descriptive statistics to examine participant characteristics in our analytic sample. Rao-Scott Chi-square tests were applied to compare categorical variables, which are presented as proportions. To examine disparities in HPV vaccination uptake, we created a composite variable with four categories based on the intersection of disability status and sexual orientation: heterosexual without disability (reference group), heterosexual with disability, LGB + without disability, and LGB + with disability. Modified Poisson regression models with robust standard errors were used to estimate adjusted prevalence ratios (aPRs) and 95% confidence intervals (CIs) for associations between HPV vaccination and this composite variable. This modeling approach was selected for its suitability in handling binary outcomes and its ability to yield interpretable prevalence ratios while accounting for overdispersion. Two main regression models were estimated: (1) a model that included only the dependent variable, the primary independent variable (the intersection of disability and sexual orientation), and survey year; and (2) a model that additionally adjusted for predisposing factors (age and race), and enabling factors (census region), alongside survey year. To explore within-group heterogeneity, we conducted additional analyses examining HPV vaccination uptake across specific disability types (sensory, cognitive, physical, and multiple disabilities), comparing each to women without disabilities. We also examined uptake across sexual orientation subgroups (lesbian/gay, bisexual, and questioning/unsure), using heterosexual women as the reference group. These stratified models used the same modified Poisson regression method and covariate adjustments as the primary analyses.

To further explore disparities in HPV vaccination patterns, we also conducted supplementary analyses to estimate aPRs for HPV vaccination uptake separately among women aged 18–26 years and 27–44 years, as well as HPV vaccine series completion among those who initiated the vaccine, using the composite disability-sexual orientation variable. All analyses were weighted to account for the BRFSS’s complex sampling design, and missing data were excluded. Statistical analyses were performed using SAS version 9.4, employing PROC SURVEY to handle both the complex sampling design and the recommended survey weights.

## Results

The analytic sample included 40,401 women aged 18–44 years. Overall, 71.5% identified as heterosexual women without disabilities, 17.3% as heterosexual women with disabilities, 7.2% as LGB + women without disabilities, and 4.0% as LGB + women with disabilities. Overall, the vast majority of heterosexual women were 27 years of age or older (84.5% of those without disabilities; 84.2% of those with disabilities). LGB + women were more likely to be younger than those who identified as heterosexual (25.0% of LGB + women without a disability and 29.1% of LGB + women with a disability). Most heterosexual women, regardless of disability status, identified as non-Hispanic White (59.7% among those without disabilities vs. 58.6% with disabilities), compared with 48.5% of LGB + women without disabilities. Across all LGB + groups, the proportion identifying as Hispanic or another racial/ethnic minority was higher than among heterosexual women. Compared with heterosexual women without disabilities, LGB + women with disabilities were more likely to be never married (56.2% vs. 32.5%), have low income (80.7% vs. 43.7%), smoke currently (32.5% vs. 12.3%), and be underweight (2.9% vs. 2.0%). They were also less likely to have completed a college degree (22.0% vs. 48.4%), possess health insurance (74.9% vs. 87.4%), have access to a primary care provider (68.9% vs. 79.5%), or have had a routine checkup in the past year (80.2% vs. 86.5%) (Table 1).

**Table 1:** Demographic characteristics of study participants (*N* = 40,401)

Unweighted Observations Weighted percent of total Characteristics	Heterosexual without disabilities *n* = 29,606 (71.5%)	Heterosexual with disabilities *n* = 6849 (17.3%)	LGB + without disabilities *n* = 2620 (7.2%)	LGB + with disabilities *n* = 1326 (4.0%)	Total *N* = 40,401	P-value
Age group						<.0001
18–26 years	15.5	15.8	25.0	29.1	16.7	
27–44 years	84.5	84.2	75.0	70.9	83.3	
Race						<.0001
White, NH	59.7	58.6	48.5	51.3	58.5	
African American/Black, NH	13.3	16.0	12.0	11.3	13.6	
Hispanic	13.2	11.2	14.7	14.4	13.0	
Other^a^	13.8	14.2	24.8	23.0	14.9	
Marital status						<.0001
Never married	32.5	37.5	52.9	56.2	35.4	
Married	54.6	37.9	34.1	24.7	49.5	
D/W/S^b^	12.9	24.6	13.0	19.1	15.1	
Education						<.0001
Less than high school	4.8	12.3	13.3	14.7	6.9	
High school graduate	20.1	29.7	23.8	29.7	22.3	
Some college	26.8	33.2	27.1	33.6	28.1	
College graduate	48.4	24.8	35.8	22.0	42.7	
Income						<.0001
< $50,000	43.7	73.1	60.7	80.7	50.9	
$50,000 or more	56.3	26.9	39.3	19.3	49.1	
Health Insured						<.0001
No	12.6	18.8	23.0	25.1	14.7	
Yes	87.4	81.3	77.0	74.9	85.3	
Have a PCP						<.0001
No	20.5	20.5	32.4	31.1	21.6	
Yes	79.5	79.5	67.6	68.9	78.4	
Routine health check^c^						<.0001
No	13.5	15.0	17.2	19.8	14.2	
Yes	86.5	85.0	82.8	80.2	85.8	
Current Smoking						<.0001
No	87.7	69.2	84.8	67.5	83.7	
Yes	12.3	30.8	15.2	32.5	16.3	
BMI						<.0001
Underweight	2.0	2.8	3.0	2.9	2.2	
Normal	58.3	702	61.3	68.7	60.9	
Obese	39.7	27.0	35.7	28.4	36.9	
Census Region						<.0001
Northeast	19.5	15.0	27.1	21.0	19.3	
Midwest	19.2	16.7	12.2	12.4	18.1	
South	47.3	58.3	47.7	56.4	49.5	
West	14.0	10.0	13.0	10.1	13.1	

*NH* Non-Hispanic, *PCP* Primary Care Provider, Values are prevalence presented as row percentages (%); Chi-square tests were used to assess differences in characteristics

Unweighted numbers and weighted percentages, adjusted for sampling weights, are reported

^a^Other included Asians, Native Hawaiians and other Pacific Islanders, American Indians and Alaska Natives, and multi-racial individuals

^b^Marital status responses included never married, married and D/W/S (divorced, widowed and separated)

^c^Routine health checkup was reported as having vs. not having routine health checkup within the last year

Behavioral Risk Factor and Surveillance System, 2014–2022, (*n* = 40,401)

**Fig. 1:**
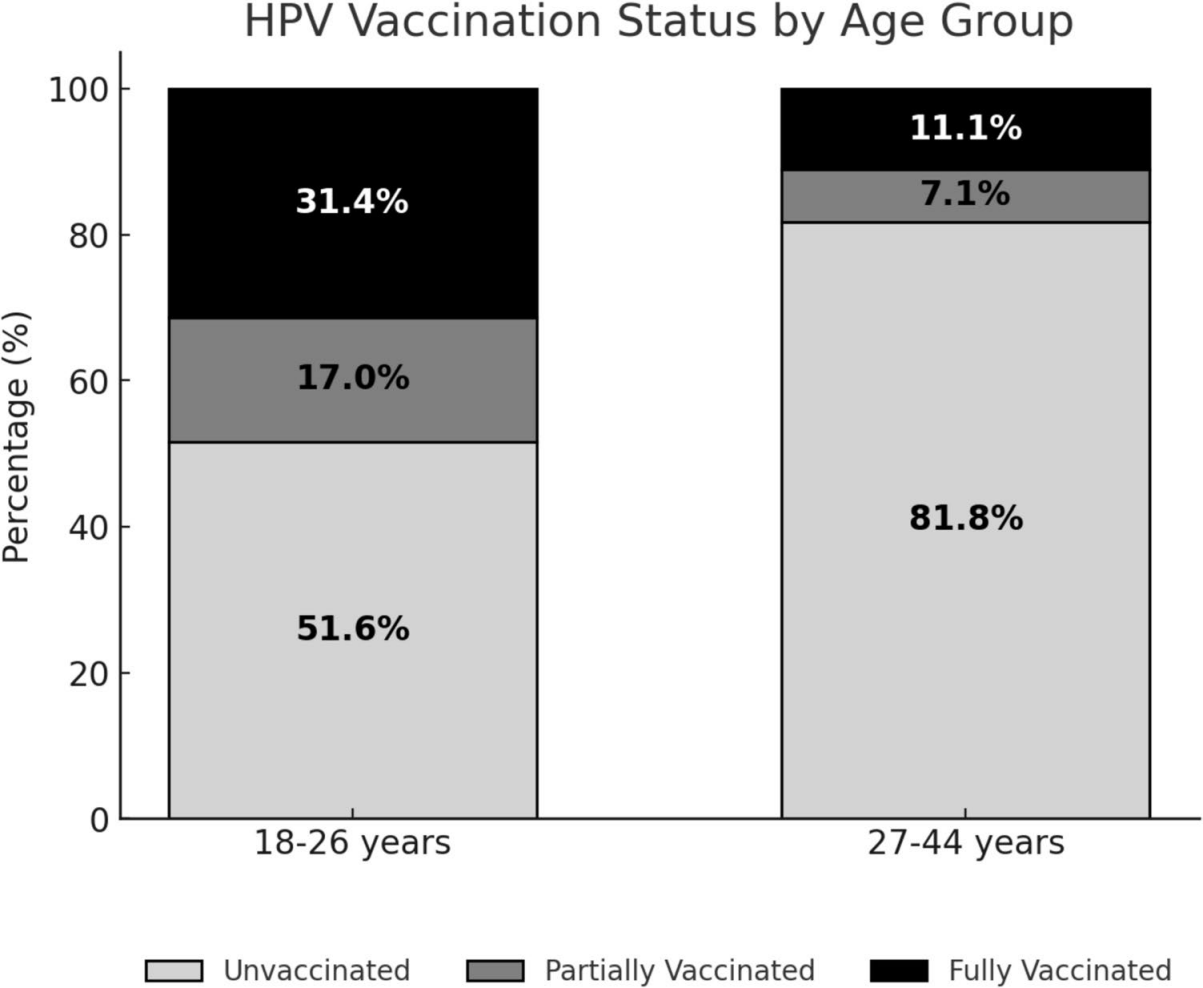
HPV vaccination series completion status prevalence by age group, among women, United States. All estimates are adjusted for sampling weights

**Fig. 2:**
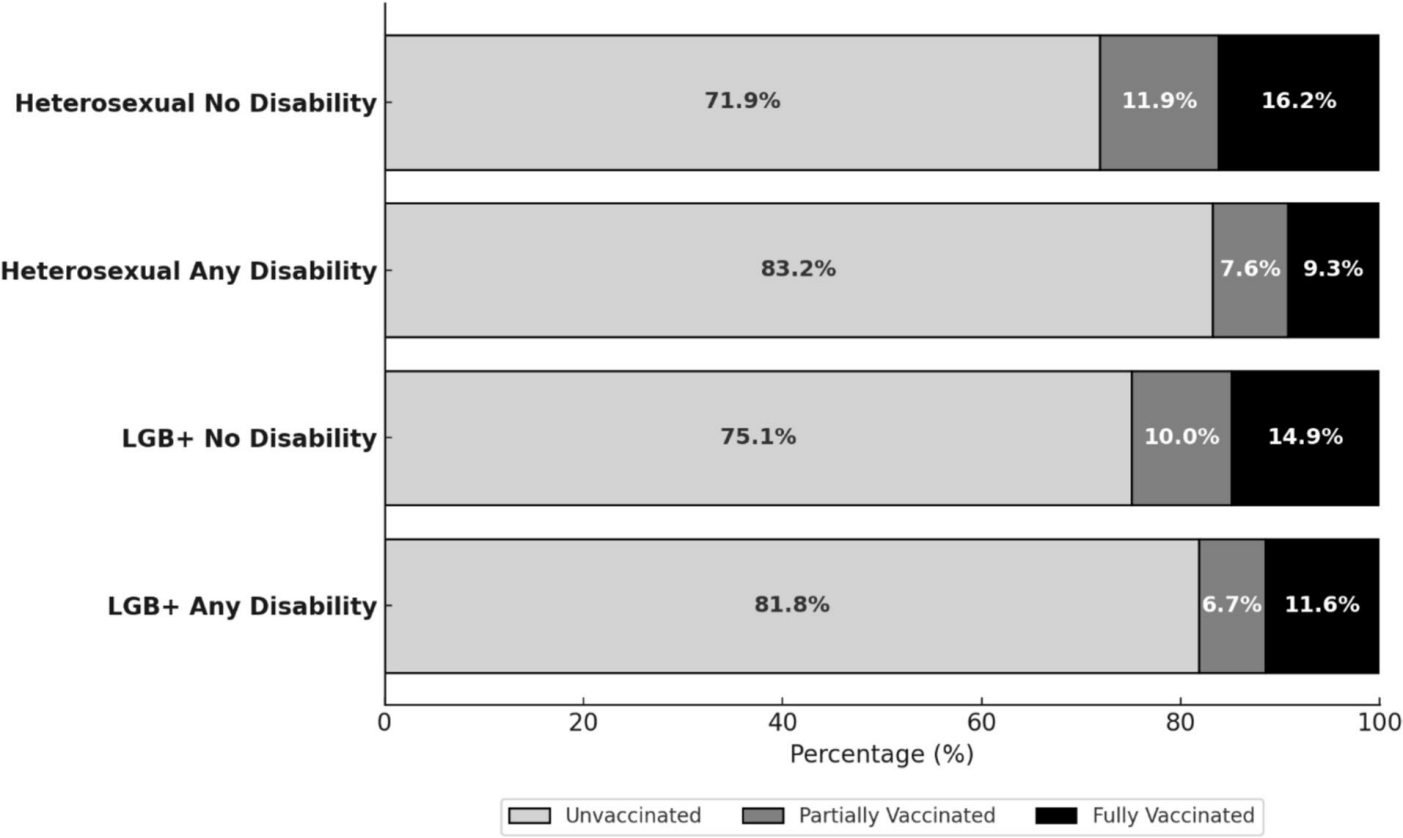
HPV vaccination series completion status prevalence by disability and sexual orientation among women, 18–44 years, United States. All estimates are adjusted for sampling weights. Source: Behavioral Risk Factor and Surveillance System, 2014–2022, (N = 40,401)

Overall, 18.8% of participants reported ever receiving an HPV vaccination, and 11.6% had completed the full vaccination series. HPV vaccination uptake was higher among younger women (18–26 years), with 48% receiving at least one dose, compared to 18% among women aged 27–44 (Fig. 1). The lowest rates of HPV uptake and series completion were observed among heterosexual women with disabilities (16.9%, 95% CI 13.0–17.8% and 9.3%, 95% CI 8.6–9.9%, respectively) and LGB + women with disabilities (18.3%, 95% CI 17.9–19.5% and 11.6%, 95% CI 11.2–12.0%, respectively), followed by LGB + women without disabilities (24.9%, 95% CI 24.3–27.5% and 14.9%, 95% CI 13.5–16.2%, respectively) and heterosexual women without disabilities (28.1%, 95% CI 26.9–28.7% and 16.2%, 95% CI 14.2–18.2%, respectively) (Fig. 2).

Women with any disability were 2% less likely to have received an HPV vaccination (aPR = 0.98; 95% CI 0.96–0.99) compared with women without disabilities. Specifically, compared to women without disabilities, those with cognitive disabilities (aPR = 0.91; 95% CI 0.79–0.98) had a significantly lower likelihood of vaccination, while those with physical disabilities (aPR = 1.02; 95% CI: 1.01–1.08) were more likely to be vaccinated (Table 2).

**Table 2:** Prevalence Ratios for Receipt of Any HPV vaccination, by Disability (*n* = 40,401)

Disability Type	Model I	Model II
	PR (95% CI)	PR (95% CI)
No disability	Ref	Ref
Any disability^**a**^	0.86 (0.83–0.89) **	0.98 (0.96–0.98) ***
Sensory disability	0.96 (0.88–0.99) ***	0.98 (0.86–1.11)
Cognitive disability	0.78 (0.68–0.88) ***	0.91 (0.79–0.98) ***
Physical disability	1.27 (1.18–1.37) **	1.02 (1.01–1.08) ***
Two or more disabilities	0.89 (0.82–0.96) ***	1.03 (1.05–1.12) ***

All models are adjusted for survey year and account for the survey’s complex sampling design. All prevalence ratios (PRs) compare each disability group to individuals with no disability (reference group)

Model–1: Adjusted for survey year

Model − 2: Adjusted for age, and race/ethnicity, census region, and survey year

^*^p < 0.01, **p < 0.001, ***p < 0.05

*CI* confidence interval; *Ref* reference category

*Source:* Behavioral Risk Factor and Surveillance System, 2014–2022, (*n* = 40,401)

^a^Findings reported from a separate regression model with Disability status as a binary outcome variable

Most groups of LGB + women, except for those who reported being questioning, were less likely than heterosexual women to have received an HPV vaccination. Overall, being LGB + was associated with a 3% lower likelihood of HPV vaccination (aPR = 0.97; 95% CI 0.94–0.98), with bisexual women 8% less likely to be vaccinated than heterosexual women (aPR = 0.92; 95% CI 0.89–0.95). In contrast, questioning women (aPR = 1.02; 95% CI 1.00–1.04) were more likely to be vaccinated than heterosexual women (Table 3).

**Table 3:** Prevalence Ratios for Receipt of Any HPV vaccination, by Sexual Orientation (*n* = 40,401)

Sexual Orientation	Model I	Model II
	PR (95% CI)	PR (95% CI)
Heterosexual	Ref	Ref
LGB + ^**a**^	0.90 (0.88–0.92) **	0.97 (0.94–0.98) ***
Lesbian/Gay	0.92 (0.88–0.96) **	0.98 (0.94–1.02)
Bisexual	0.81 (0.78–0.83) **	0.92 (0.89–0.95) **
Questioning/Unsure	1.02 (1.01–1.06) ***	1.02 (1.00–1.04) ***

All models are adjusted for survey year and account for the survey’s complex sampling design

Model–1: Adjusted for survey year

Model − 2: Adjusted for age, and race/ethnicity, census region, and survey year

^*^p < 0.01, **p < 0.001, ***p < 0.05

*CI* confidence interval, *Ref* reference category

Behavioral Risk Factor and Surveillance System, 2014–2022, (*n* = 40,401)

^a^Findings reported from a separate regression model with sexual orientation as a binary outcome variable

In both unadjusted and adjusted models, heterosexual women with disabilities and LGB + women with disabilities were significantly less likely to report receiving an HPV vaccination than heterosexual women without disabilities, reflecting disparities at the intersection of disability and LGB + status. Specifically, after adjusting for age, race/ethnicity, census region, and survey year, heterosexual women with disabilities were 5% less likely (aPR = 0.95; 95% CI 0.93–0.97), LGB + women with disabilities were 3% less likely (aPR = 0.97; 95% CI 0.96–0.98), and LGB + women without disabilities were 2% less likely (aPR = 0.98; 95% CI 0.95–0.99) to be vaccinated compared with heterosexual women without disabilities (Table 4).

**Table 4:** Prevalence ratios for receipt of any HPV vaccination, by disability and sexual orientation (*n* = 40,401)

Disability and Sexual orientation	Model I	Model II
	PR (95% CI)	PR (95% CI)
Heterosexual without disability	Ref	Ref
Heterosexual with disability	0.88 (0.85–0.93)**	0.95 (0.93–0.97)***
LGB + without disability	1.02 (1.01–1.09)*	0.98 (0.95–0.99)***
LGB + with disability	0.92 (0.89–0.95)**	0.97 (0.96–0.98)***

All models are adjusted for survey year and account for the survey’s complex sampling design

Model–1: Adjusted for survey year

Model − 2: Adjusted for age, and race/ethnicity, census region, and survey year

^*^p < 0.01, **p < 0.001, ***p < 0.05

*CI* confidence interval, *Ref* reference category

Behavioral Risk Factor and Surveillance System, 2014–2022, (*n* = 40,401)

Among participants who initiated the HPV vaccination series, a similar pattern emerged for completion of the full series. Compared with heterosexual women without disabilities, heterosexual women with disabilities were 12% less likely (aPR = 0.88; 95% CI 0.83–0.93) to complete the full series. Similarly, LGB + women with disabilities (aPR = 0.90; 95% CI 0.83–0.98) and LGB + women without disabilities (aPR = 0.94; 95% CI 0.88–0.99) were less likely to complete the series than heterosexual women without disabilities (supplementary Table 2).

In age-stratified analyses, with the exception of heterosexual women with a disability, there was less evidence of disparities in HPV vaccination among younger women (18–26 years) than in the older group. However, even in this age group, heterosexual women with disabilities were 7% less likely (aPR = 0.93; 95% CI 0.82–0.97) to be vaccinated than heterosexual women without disabilities. In contrast, among women aged 27 to 44, those who had disabilities and LGB + women were less likely to be vaccinated than heterosexual women without a disability. For example, LGB + women with disabilities were the least likely (aPR = 0.95; 95% CI 0.93–0.98) to be vaccinated compared with heterosexual women without disabilities (Table 5).

**Table 5:** Prevalence Ratios for Receipt of Any HPV Vaccination, by Disability and Sexual Orientation Among Women, Stratified by Age (18–26 and 27–44 Years)

Women aged 18–26 years (14,310)		
Disability and Sexual orientation	Model I	Model II
	PR (95% CI)	PR (95% CI)
Heterosexual without disability	Ref	Ref
Heterosexual with disability	0.94 (0.86–0.99) ***	0.93 (0.82–0.97) ***
LGB + without disability	0.96 (0.88–1.06)	0.97 (0.87–1.08)
LGB + with disability	1.03 (0.91–1.15)	1.01 (0.90–1.37)

Women aged 27–44 years (*n* = 26,091)		
Heterosexual without disability	Ref	Ref
Heterosexual with disability	1.01 (0.98–1.02)	0.98 (0.97–0.99) ***
LGB + without disability	0.94 (0.92–0.97) **	0.97 (0.95–0.99) ***
LGB + with disability	0.91 (0.88–0.95) **	0.95 (0.93–0.98) ***

All models are adjusted for survey year and account for the survey’s complex sampling design; Younger adults in this study are those within the age range of 18 to 26 years

Model–1: Adjusted for survey year

Model − 2: Adjusted for race/ethnicity, census region, and survey year

^*^p < 0.01, **p < 0.001, ***p < 0.05

*CI* confidence interval, *Ref* reference category

Behavioral Risk Factor and Surveillance System, 2014–2022, (*n* = 40,401)

## Discussion

Cervical cancer remains a significant public health concern, particularly among adult women, despite the widespread availability of effective HPV vaccines for nearly 20 years. As a primary prevention strategy, HPV vaccination has the potential to significantly reduce the incidence of cervical cancer. However, cost and access barriers continue to limit HPV vaccination uptake, particularly among minority populations [39–41]. While the incidence of cervical cancer among women aged 20–24 declined between 2012 and 2019, corresponding with the first patient cohorts eligible for HPV vaccination in childhood, the rate among women aged 30–44, who were ineligible for early vaccination, increased by 2% annually during the same period [41–43]. An estimated 39% of cervical cancer cases are diagnosed before age 45 [41, 43] highlighting a critical window for prevention efforts.

This study provides two key empirical contributions to the literature. First, by using a nationally representative dataset with comprehensive measures of both sexual orientation and disability, it offers novel insights for understanding disparities in HPV vaccination uptake at the intersection of these social identities. Second, it examines uptake among two distinct age groups: young adults (18–26 years), who were eligible for routine HPV vaccination, and mid-adults (27–44 years), who were not eligible for childhood vaccination but qualified for catch-up vaccination.

Our findings suggest a low overall prevalence of HPV vaccination uptake (18.7%) and series completion (11.6%) among women in the study, highlighting a significant gap in efforts to prevent HPV infection and cervical cancer. Unlike most cancer prevention strategies, which focus on secondary or tertiary prevention (e.g., screening and treatment), the HPV vaccine is a rare example of primary prevention, directly reducing the risk of HPV-related cancers before they develop. However, our results indicate that many women are missing this critical opportunity for prevention, leaving them vulnerable to cervical cancer. We found that HPV vaccination and series completion rates were higher among adult women aged 18 to 26 years compared to adult women aged 27 to 44 years, a pattern consistent with prior research [12]. This trend is expected, as younger individuals are closer to the recommended age for HPV vaccination and may have benefited from stronger provider recommendations and broader public health campaigns. In contrast, women aged 27 to 44 years, who fall into the catch-up vaccination category, may have faced barriers such as fewer provider recommendations, lower perceived risk, and limited awareness of vaccine benefits [44–46]. These findings demonstrate the need for tailored interventions to improve HPV vaccine uptake among women aged 27 to 44 years [47] ensuring that all eligible individuals have equitable access to primary prevention strategies against HPV-related cervical cancer.

LGB + women are at risk for HPV infection and cervical cancer [20] yet our findings indicate they are slightly less likely than heterosexual women to report HPV vaccination. Importantly, substantial variation exists within LGB + groups. For instance, HPV vaccination uptake was lower among bisexual women compared to heterosexual women and other LGB + subgroups. These findings align with prior research showing lower HPV vaccination rates among bisexual women relative to heterosexual women [48]. This trend is particularly concerning given evidence that bisexual women may have a higher prevalence of HPV infection than both heterosexual and women identifying as lesbian/gay [20]. A potential explanation for the lower HPV vaccination uptake among bisexual women is their limited visibility within both heterosexual and LGBTQ + communities [30] which may result in reduced provider recommendations and support for vaccination [49]. Additionally, bisexual women may face greater health care discrimination or bias, leading to lower perceived importance of vaccination or delayed access to preventive healthcare services [49].

Disability status has often been underrepresented in studies examining HPV vaccination disparities, making our findings a significant contribution to the literature on HPV and cervical cancer prevention for women with disabilities. After adjusting for key covariates, our study found that women with any disability were less likely to report receiving the HPV vaccination. This trend was particularly pronounced among women with cognitive disabilities. One potential explanation for this could be the barriers to healthcare access commonly faced by women with disabilities, including inaccessible public health communications (e.g., lack of plain language resources), reductions of disability-related supports, services, and accommodations (e.g., lack of home care, or sign language interpreters), which may prevent them from receiving preventive care such as HPV vaccinations [50]. Additionally, cognitive disabilities may be associated with challenges in navigating the healthcare system, including understanding vaccine recommendations or engaging in shared decision-making with healthcare providers [19]. These disparities may also be driven by deeply rooted social attitudes. Women with disabilities are frequently perceived as asexual, celibate, or childlike, assumptions that may lead healthcare providers to overlook or deprioritize their need for sexual and reproductive health services, including HPV vaccination, cervical cancer screening, and HIV prevention [19, 24, 33, 34]. Such biases can result in fewer provider recommendations and missed opportunities for preventive care. Addressing these stigmatizing perceptions through provider training and inclusive care practices is essential for improving access and reducing inequities in vaccination and broader sexual health outcomes.

Interestingly, women with physical disabilities and those with multiple disabilities reported higher HPV vaccination receipt, which may reflect greater healthcare engagement, possibly due to increased contact with healthcare providers, frequent medical visits, or targeted health interventions. It may also reflect the role of healthcare providers’ proactive measures in addressing multiple health concerns, which could extend to offering HPV vaccination as part of routine care. Supplementary analyses showed that these women were more likely to have a primary care provider and receive routine checkups, suggesting increased contact with the healthcare system. However, they were less likely to have health insurance, highlighting potential barriers to continuous healthcare access despite frequent medical interactions.

Intersectionality improves our understanding of how various dimensions are at play in shaping and influencing social positions and power relations [51, 52]. Guided by intersectionality, this study provides novel information on HPV vaccination uptake across both disability status and sexual orientation identity at the population level. Our findings contribute to the scientific literature by identifying heterosexual women with disabilities, whose healthcare experiences are shaped by ableism but not heterosexism, as a particularly underserved group. LGB + women with disabilities, who experience both ableism and heterosexism, had the second lowest prevalence of HPV vaccination receipt and series completion, followed by LGB + women without disabilities, who navigate heterosexism but not ableism. After adjusting for key covariates, heterosexual women with disabilities, LGB + women with disabilities, and LGB + women without disabilities were all less likely to report HPV vaccination uptake compared to heterosexual women without disabilities. However, heterosexual women with disabilities were the least likely to be vaccinated and complete the full series, suggesting that, contrary to our hypothesis, disability and sexual orientation may not have a compounding effect on HPV vaccination rates, despite the observed lower prevalence among LGB + women with disabilities. This also suggests that disability may have a stronger association with HPV vaccination uptake than sexual orientation. However, it is important to note that both disability and sexual orientation likely serve as proxies for underlying factors, such as health behaviors, access to care, and systemic discrimination, which may more directly influence HPV vaccination uptake.

Among those who initiated the vaccine series, heterosexual women with disabilities were less likely to be fully vaccinated compared to heterosexual women without disabilities. This difference may be explained by potential barriers such as limited access to healthcare, concerns regarding vaccine efficacy, or challenges in managing healthcare appointments due to disability-related factors [19].

Another subgroup identified as needing special attention is reproductive-aged women with disabilities who did not receive HPV vaccination in childhood. Our findings suggest that disparities in HPV vaccination coverage are fewer among younger LGB + women, possibly because routine vaccination occurs during childhood and early adolescence, before many individuals begin to explore or disclose their sexual orientation, which may reduce the impact of stigma or provider bias that older LGB + individuals might face when seeking vaccination [56, 57]. In contrast, pronounced gaps remain among women above the age of 26, particularly among those with disabilities. This is likely because they did not benefit from early HPV vaccination campaigns and must rely on catch-up efforts [44–46]. These targeted catch-up strategies are important for ensuring that older cohorts, who were ineligible for routine vaccination recommendations during adolescence, have a greater opportunity to receive the HPV vaccine and reduce their future risk of cervical cancer.

The U.S. Department of Health and Human Services has set a goal to ensure 80% of eligible individuals receive the HPV vaccine by 2030 [53]. Achieving this goal requires targeted interventions, including tailored education, culturally sensitive communication, and provider training to address the unique needs of older women, heterosexual women with disabilities, and LGB + women with disabilities. Outreach efforts must be inclusive, recognizing the diversity within these groups to ensure they have the necessary support to make informed decisions about vaccination. Addressing these disparities is essential for increasing vaccination rates and reducing the burden of HPV-related cervical cancer. To eliminate cervical cancer as a public health threat, equitable vaccine access must be ensured, particularly for underserved populations. This requires policy reforms and a coordinated effort from healthcare systems, community organizations, and policymakers to foster an inclusive, accessible healthcare environment for all women.

### Limitations

This study has several limitations. First, BRFSS is not nationally representative for analyses involving sexual orientation, as not all states administer the optional SOGI module. Therefore, our findings should be interpreted in the context of the 20 participating states and may not be generalizable to the U.S. population as a whole. Second, to avoid small sample sizes in intersectional subgroups, we combined certain sexual orientation and disability categories, which may have obscured unique patterns within these specific subgroups. Specifically, we grouped respondents who selected ‘something else’ as their sexual orientation with lesbian, gay, or bisexual women. While this approach is consistent with prior public health research, and the ‘something else’ reflects non-heterosexual identity consistent with sexual minority identity (e.g., queer), it may obscure differences between groups and limit the precision of our estimates for individuals with less commonly reported sexual identities. Third, since the survey relies on self-reported data, responses are subject to reporting errors, particularly underreporting of the outcome. Although self-reported vaccination status has been shown to be reasonably reliable, [54] accurately determining whether participants received the HPV vaccine can still be challenging. Fourth, we restricted our sample to individuals who identified as ‘female’ and had not undergone a hysterectomy, focusing on those who presumably have a cervix. In addition, we excluded respondents who identified as transgender. While BRFSS includes a question on transgender identity, it has not consistently collected information on sex assigned at birth, which limits the ability to accurately identify gender-diverse individuals who may have been assigned female at birth. As such, our analysis is limited to cisgender women and does not capture disparities among transgender and nonbinary populations, who are also eligible for and in need of HPV vaccination but often face unique barriers to care. Fifth, all data were cross‐sectional, limiting our ability to establish temporality for some associations. Nonetheless, sexual orientation, like many disabilities, including cognitive disabilities, is often determined or recognized early in life and likely precedes factors such as educational status, income and availability of health insurance. However, in other cases, these factors may increase the risk that a woman develops a disability. Therefore, there may be residual confounding. However, without longitudinal data, we cannot confirm the temporal relationship.

In addition, although factors such as limited access to health insurance and provider bias may mediate the observed associations, our study was not designed to examine these structural determinants in depth. Furthermore, only a few states included the optional HPV vaccine module in any given year, limiting our ability to explore geographic variations in vaccination.

Finally, as the BRFSS data collection procedures were modified in 2020 due to the COVID-19 pandemic, there may have been shifts in HPV vaccination rates due to pandemic-related disruptions in healthcare access and preventive services. Nevertheless, BRFSS remains a reliable and nationally representative data source. Despite these limitations, this work contributes to the literature on HPV vaccinations and highlights the need for increased vaccination uptake and completion across all population groups. To date, no other study has conducted an intersectional analysis of HPV vaccination among younger (18–26 years) and later reproductive-aged women (27–44 years). These findings represent an important step to understanding differences in HPV vaccination across the intersection of disability status and sexual orientation.

## Conclusion

The HPV vaccine is the first and only vaccination that directly prevents cancer, yet uptake remains strikingly low among all populations. This study provides important insights into disparities in HPV vaccination uptake, highlighting groups of women who are least likely to receive the vaccine: heterosexual women with disabilities and reproductive-aged women not vaccinated in childhood and adolescence. Overall, HPV vaccination and completion rates remain low. Notably, women aged 18–26 years were more likely to be partially or fully vaccinated compared to women aged 27 to 44 years. This is particularly concerning given that HPV infections that cause cancer are more prevalent among adults aged 27–45 years [55] and that 39% of cervical cancer cases are diagnosed before age 45 [41]. These findings highlight the need for enhanced efforts to improve primary prevention through HPV vaccination across all groups. They also emphasize the critical importance of catch-up vaccination for women older than 26 years, who did not benefit from adolescent vaccination recommendations.

Our intersectional analysis uncovered previously hidden disparities, showing that disability status is particularly important in predicting HPV vaccine uptake. Heterosexual women with disabilities and LGB + women with disabilities were less likely than heterosexual women without disabilities to initiate and complete the vaccine series. Furthermore, age-based analyses revealed pronounced gaps among younger heterosexual women with disabilities and older LGB + women with disabilities.

Addressing these disparities requires targeted programs that ensure all eligible women, regardless of disability status or sexual orientation, receive and complete HPV vaccination, a key step toward achieving health equity. Structural and policy-level interventions are needed to improve healthcare access, reduce stigma and implicit bias, and ensure inclusive, accessible vaccination services. From a life-course perspective, robust provider recommendations and accessible vaccination programs can further narrow gaps among younger women, while older women, especially those with disabilities, require proactive catch-up strategies. By recognizing how intersecting social identities and age shape HPV vaccination disparities, public health efforts can be tailored to ensure that all women, regardless of disability status, sexual orientation or age, have the opportunity to benefit from HPV vaccination and reduce their risk of HPV-related cervical cancer.

## Declarations

### Data availability

No datasets were generated or analyzed during the current study.
